# The Asylums of Italy, Germany, and France, &c.

**Published:** 1858-10-01

**Authors:** J. T. Arlidge


### Art. V.—THE ASYLUMS OF ITALY, GERMANY, AND FRANCE, &c.

BY J. T. ARLIDGE, M.B., A.B. (LOND.)

### NOTES ON SOME OF THE ASYLUMS IN FRANCE.

The very excellent and interesting Notes, by Dr. Webster, on many of the public asylums of France, especially those of its northern half, have made the readers of the "Psychological Journal" acquainted with the system of treatment and management, and the principles of construction generally pursued in that country; there remain, however, several asylums in its southern and eastern parts hitherto undescribed, yet well worthy of examination and description.

The French law requires, as pointed out by Dr. Webster, in his first contribution to this Journal (Vol. III. 1850, p. 530), each department to provide an asylum for the reception and treatment of its insane paupers; or to arrange, under the sanction of the Minister of the Interior, with a public or private asylum in the same or a neighbouring department for their reception; or, in certain cases, to appropriate a separate division to them in civil hospitals, provided there be sufficient accommodation for not less than fifty patients. For further information on the French laws affecting asylums and their administration, we would, to avoid unnecessary repetition, refer the reader to Dr. Webster's Notes, particularly to the page above quoted, and to Vol. V. p. 376.

The departmental authorities have availed themselves, in providing for their insane poor, of each of the three modes just now cited. In several departments buildings specially constructed for the reception and treatment of the insane have of late years been constructed; in others, again, ancient religious houses and hospices for the poor have been converted to the purpose; whilst in others some portion of the general hospitals of their principal towns affords the only provision for their lunatics. Besides public departmental and strictly private asylums, there are others scattered through France belonging to religious orders, and under their direction—for instance, those at Lyons and Bourg, appertaining respectively to the Brethren of St. Jean de Dieu, and the Sisters of St. Charles.

Of the several plans for administering to the wants of the insane, that of placing them in the wards, or in a wing of a general hospital, is the worst. It inevitably entails all the evils of a bad site in the midst of a large town, a less pure air, a state of more or less complete imprisonment, enclosure by walls and buildings, the deprivation of all cheerful views, the contiguity of noises, the want of space for exercise and outdoor employment, defective constructional details, and the absence of that autocracy and unity of management which can be attained only in an independently built and organized asylum under one head.

This plan appears to have been almost universal in France; the insane, so far as they were looked after at all, were brought together mostly into the hospital or the hospice of the town, and there stowed away in the highest, the lowest, or the most out-of-the-way rooms, wards, or vaults, where their fellow-creatures might be least offended by their sight or sound. Some found their abiding place in prisons, where, if possible, their state must have been even more deplorable than that of their fellow-sufferers in the hospitals. It is not, however, our intention to write the history of the French asylums as they were. Esquirol, in the second volume of his great work ("Des Maladies Mentales," 1838), gives an able sketch of their condition at the time he wrote, from which some conception may be formed of the miserable condition of the insane in France, although considerable ameliorations then had been already effected.

Although the system of confining lunatics in particular wards, or in the wings or detached buildings of general hospitals, still prevails in France, and, to the disgrace of those large and opulent towns of Lyons and Montpelier, yet we are happy to add, that its evils are everywhere recognised, and that asylums in the country are proposed to be erected in the place of the ill-suited town establishments. Unfortunately, the foreign wars in which France has been engaged, the domestic troubles which have overtaken her, the recent failure of one great source of her wealth by the vine disease, her laws of property and inheritance, and her large armies, have, whilst increasing the number of her dependent citizens, also increased taxes, and thereby have put it out of the power of the central and departmental authorities—at least in many districts—to proceed in that career of reform in asylum construction and management which Pinel and Esquirol originated, and Ferrus and so many other worthy followers have promoted. Nevertheless, France can now point to not a few most admirably-constructed asylums, from which our architects might learn some useful lessons—one at least of no little importance—viz., that our stereotyped system of wards is not essential in asylum building, but rather a serious error, which cannot be too soon confessed and given up.

There is one feature, which, as common to almost every French public asylum, deserves notice here, viz., that pauper and paying patients are received into the same institution; *i.e.*, to use a common phrase, they are "mixed asylums." The paupers are provided for by a general rate; the paying patients are subdivided into classes, according to the sums paid for their maintenance.

We will not at present extend these general remarks to greater length, but proceed with our notes on the construction and management, medical and moral, of those asylums which we had the opportunity of visiting; premising only that the amount of information to be given is varied by the circumstances of the visit, the time permitted for it, the facility for inquiring into details, the opportunity for collecting statistics, the character of our conductor, who at times was the chief, at others the second physician, and at others, again, only an "interne;" still, in all cases, we would most willingly acknowledge the kindness and courtesy everywhere received, and the readiness with which information was imparted. And, first, of—

THE ASYLUM AT MARSEILLES.

For several ages (since 1600, A.D.) the insane and other sick persons unsuited to the general hospitals of the city were placed in a building by themselves; and, after many vicissitudes, were collected in the Hospital of St. Lazare, previously a house for lepers, situated in the northern suburb, on the road to Aix. In this place, which Ferrus calls a disgrace to the city, they remained until the new asylum, about to be described, was opened for their reception, in 1835.

So long ago as 1823, the resolution was taken to build another asylum; and a premium having been offered for the best plan, that of M. Pinchot, the architect of the department, was selected. M. Esquirol was consulted respecting the plan, and inspected it and the proposed site in 1824; but matters were not definitively settled until 1830, when political events again interfered to prevent the plan being carried out, and it was not before 1833 that the construction was proceeded with. It was calculated to provide accommodation for 300 patients. In the course of the next eighteen years, the accumulation of patients and the demands for admission were so large, that a large addition to the building became imperative, and, in January, 1851, a new wing was commenced, consisting of five divisions, and capable of holding 340 inmates; and many important modifications of the original structure were proceeded with. At the period of my visit, in 1854, other alterations and additions were in progress, and little of the original building designed by M. Pinchot, under the auspices of Esquirol, remained, except the section appropriated to the residences and offices of the director and other officers.

The asylum is situated about a mile and a half out of Marseilles, beyond the Plaine St. Michel, in the Avenue St.

Pierre. It is placed in a valley; the surrounding hills are bold and varied in outline, and the country has much beauty. It serves for two departments—that of the Bouches-du-Rhone, and that of the Var—and, at the date of my visit, contained above six hundred patients, all of them paupers; the intention, however, was, so soon as accommodation could be provided, to receive also pensioners.

It is built of stone, found abundantly in the neighbourhood, and is everywhere of only two floors. Its principle of construction is that very much pursued in France, which Esquirol so ably advocated—viz., a group of buildings surrounding a square court, constituting hollow squares, and furnished with internal and external corridors. These hollow squares are arranged parallel on either side a central court, in the middle of which are placed the kitchen, scullery, baths, boiler-room, &c., and closing it in front, the chapel and corridor leading from it on each side; behind, this central court is open to the garden, although some feet above it, owing to an artificial elevation of the ground for the proper disposition of the several structures. In advance of the chapel is a small court, surrounded on three sides by the buildings appropriated to the officers, and having a corridor running around it. There is also an open corridor of communication, extending backwards from the official residences, the entire length of the wings forming the two sides of the central court; and from this again, on each side, a short corridor communicates with the kitchen.

The general disposition of the accommodation is that of sitting, eating, and work-rooms on the ground floor, and dormitories above; in the divisions for the refractory, dirty, and epileptic, this rule is departed from by the construction of sleeping-rooms on the ground floor. No general system of lighting or of warming and ventilating is in operation. For day use there are a dining-room, an unfurnished room called a *chauffoir*, and a work-room for each division except the refractory, which has only a refectory or dining-room.

On entering, from the official residences, the corridor of communication between the several sections of each half of the institution, the first room reached is the visiting-room, in which the patients see their friends, who are allowed to visit once a week. It is a moderate-sized, comfortable room, furnished with tables and chairs, and its walls papered. Continuing along the corridor, we come to a door which opens in the centre of one of the divisions by a small hall, from which a staircase ascends in front to the first floor, whilst a door opens on one side into the work-room, on the other into the day-room (*chauffoir*). The dining-room is placed at right angles to the rooms just named, and can be entered at one corner from the corridor, or, at its centre, from the enclosed court belonging to the division.

On the female side are two large work-rooms, furnished with many small tables, around which the patients were grouped in small parties, engaged in needlework and similar occupations. The attendant sat at one end, with a table and desk before her slightly elevated. The only attempt at ornamentation in these rooms consisted of a crucifix, with some other religious emblems and artificial flowers placed against the wall above the attendant's chair. On the male side the patients were employed in shoemaking, tailoring, and carpentry: the larger number in the two first occupations.

The day-room, or *chauffoir*, of each division is intended especially for use when the weather is unfavourable to out-door exercise. It derives its particular name from the presence of a stove in its centre, and is, in all other respects, little suggestive of comfort, and devoid of inducements to use it, inasmuch as, with the exception of a few benches, it is a bare, unfurnished room, saving what the religious fervour of some of the patrons of the establishment may supply in the shape of some saintly portrait and a crucifix. Except these *chauffoirs*, there is no room for the assemblage of the inmates to partake in general amusements.

The refectory, or eating-room, is only used at meal time, and, excepting the fixed tables and benches, is equally destitute of furniture with the day-room. In the wards for the clean and orderly, the dining-tables are made of a polished marble slab, supported on iron legs fixed to the floor. In the other wards they are of wood, but covered with figured oilcloth, and irremovable, which gives the advantage of a smooth, polished surface, easily cleansed. The walls here, as in the rooms generally, are plastered and coloured.

The windows on the ground floor all look inwards upon the enclosed court. In construction they follow the usual French fashion, and are of sufficient size, but everywhere barred with upright iron bars. Externally, also, are strong Venetian shutters (*Persiennes*), which seemed to be everywhere closed; indeed, so far as I could judge, they were rarely opened. Happily this practice deprived the inmates of no prospect, for the view of an enclosed court is not exhilarating, and cannot in justice be called a prospect. Moreover, any one acquainted with the sunny sky, the heat, and bright light of the south, will see some good reason for darkening the rooms which the dweller in our mirky, misty atmosphere could not surmise. Yet, withal, this shutting out of patients from external influences, and from the cheering face of nature, is a disadvantage and an evil, whether arising from an unnecessary closure of shutters, or from the inevitable consequences of the faulty system of construction of enclosed courts.

The unpleasant appearance of bars and closed *Persiennes* was somewhat relieved by curtains and their appendages on the inside, furnished to the sitting-rooms down-stairs, and to some of the dormitories above.

One side of the block of buildings occupied by the dirty and epileptic patients—the ground floor—is devoted to dormitories, the floors of which are tiled, and unfurnished with carpet. The beds are placed along the entire length of the room, leaving a central avenue, two or three inches lower than the rest of the floor, and bordered on either side by a stone edging. Those of our readers who have visited the Salpêtrière, at Paris, will remember that a similar arrangement prevails there in some wards. What its purpose may be I cannot conceive, although its disadvantages are sufficiently evident,—for it forcibly recalls the arrangement of a stable-floor, and must frequently endanger a twisting of the ankle, a false step, or a stumble. The division for the refractory and noisy is the most remote from the central offices: it contains about twenty-four single rooms, placed between the open corridor on one, and an enclosed corridor on the other side, around two sides of a hollow square. On a third side was the common eating and sitting-room. The enclosed court was assigned to the patients for exercise; it was a bare, unpaved yard, without flower-beds. The corridors were floored in this, as in the other divisions, with asphalte. Each single room, or cell, was furnished with a bed fixed to the floor, and, in one corner, for the wants of the patient when confined in it, with a thick stone, hollowed out like the seat of a water-closet, having a utensil within the enclosed space beneath, which could not be reached except by a small door opened from the corridor outside, under the charge of the attendants. The cells were spacious and lofty, about 13 feet square; their window was placed high up, in size about 2½ by 2 feet, without frame and unglazed, but fitted with three upright iron bars, and externally with a strong shutter having a spring-bolt and a drop-bar, and shut or opened from the corridor.

The doors opened outwards against the wall, and besides a lock, were likewise furnished with heavy bolts. An opening about 9 inches square, closed by a door, served for the purpose of inspection. As the doors were flush with the wall externally, there was necessarily a recess within the room, between the door-jambs, equal to the thickness of the stone wall, and being bounded by acutely cut angles, threatened danger to the patient or attendant in any possible struggle during ingress or egress, and a means of doing serious mischief to a patient so inclined.

The walls of these rooms were whitewashed, and the floors were of asphalte or stone. No padded room existed.

At the top of the stairs, leading from the ground floor, a dormitory opens right and left, the attendants' rooms being interposed. On the stair-landing were a stone sink, two taps for cold water, and a store-closet. Except those taps (*robinets*), no provision was made for ablution, whether in the dormitories or about the sitting-rooms beneath. The side of the attendants' room, abutting on the dormitory, was fitted with a small window for supervision,—a provision, however, of no great utility or importance where, as in this case, the apartment is used only at night, unless, indeed, the attendants are made watchers, and required to keep awake during the night—an obligation not to be imposed upon those who have borne the burden of the day. A moment's practical consideration will remove any impression which may be entertained theoretically of the advantages of inspection windows overlooking dormitories. For, supposing a sufficient light is burnt in the sleeping-room to make every movement visible, we are dealing with sleeping and sleepy attendants covered up in bed, deep in slumber from the day's fatigue, and indulging, it is to be hoped, in an oblivion of lunatics and lunacy. To expect such to be constantly peering through this window, or to be aroused by any rustle or movement going forward, is to expect something contrary to human nature, and to impose a degree of responsibility where it cannot be undertaken. Again, it is usual to glaze such windows—a practice which directly frustrates, in a great degree, their object, by opposing the transmission of sound; and the chance is, especially in the case of the nurses, that, for privacy sake, the window is screened by a blind, and the practicability of overlooking the ward completely marred. On the other hand, the existence of the window may be said, in the accepted phraseology of the day, to have a moral influence upon the patients, by favouring the notion of constant supervision of their movements; a conceivable influence and unobjectionable, if only it does not induce their guardians to attribute a positive utility to the means of exerting it, so far as to place confidence in it to the neglect of efficient measures.

However, to return from this digression, we are glad to say that at Marseilles this theoretical way of exercising nocturnal espionage over the inmates of the dormitories was not solely relied upon, but that a night-watch was provided, two attendants being set apart in each department of the asylum for that purpose—one being constantly on duty in the infirmary, whilst the other perambulated the several dormitories, which are partially illuminated by a small lamp in each.

Except the beds and a night-commode, the dormitories had no other furniture. Their floors were tiled, and the walls white-washed. Tiled bed-room floors strike an English traveller as cold and comfortless in appearance; but the natives of South France are accustomed to no other, and do not even look for the strip of carpet by the bed-side, which the hotel proprietor, pandering to the luxury of the English, is wont to supply.

The dormitories have windows on two sides, but those on one side have been constructed nine feet from the floor, and only about two feet six inches square. This faulty arrangement is, however, to be abolished, and the windows enlarged to the same dimensions as those in the opposite wall, which are in every respect satisfactory, except in being protected (?) externally by iron bars.

The bedsteads, placed against the opposite walls of the dormitories, so as to leave a central space from one end of the room to the other, are for the most part of iron: some wooden trough-bedsteads or cribs remained, but were in course of progressive removal. The bottom of the bedsteads was about two feet from the floor; on this was placed a straw paillasse a foot thick, and upon this, again, a flock bed; consequently the patient was considerably elevated above the ground. The further bedding consisted of a pair of sheets, a woollen blanket or coverlet, and a coloured cotton counterpane. In mischievous cases, or for extra warmth, quilted coverlets were put into use. The bedsteads in the epileptic dormitories were lower than those elsewhere; but in all other respects the same. In the case of dirty patients the flock bed was omitted, the patient sleeping on a bag of straw, contained in a sort of iron box constructed in the bedstead, and having beneath it an iron drawer to receive the urine which percolates through.

The infirmary for casual sick cases and paralytics was on the first floor, and differed in no respect from one of the dormitories, except that it had a few more beds in it,—viz., twenty or twenty-four, and that some attempt was made at ornamentation. No special form of bedstead or bed was in use to meet the exigencies of particular cases. A useful adjunct to the infirmary was added, in the shape of a small room opening directly from it, and furnished with a stove to heat the ptisans and drinks for the sick, and with other conveniences to provide for their varying necessities.

In the infirmary alone, of all the sleeping apartments, was there any plan for warming: this consisted in the economical use of the heated air from the stove of the room (the *chauffoir*) below, which passed from an air-chamber surrounding the furnace, through a wide flue, to the floor of the infirmary, and entered the room through a brass grating. This plan was said to succeed, and to supply sufficient warmth.

A bath-house (*salle des bains*) was provided for each half of the establishment, and consisted of a distinct building, having three apartments, all lighted from above:—1. A waiting or dressing-room; 2. A bath-room for clean patients; and 3. One for the dirty. The baths, six in number, are arranged in a circle, leaving a central space occupied by the mains, and the hot and cold water-pipes proceeding from them, belonging to the several baths. At this part the water, hot and cold, was turned on or off. The baths are of copper, tinned, encased in wood, and severally furnished with a wooden lid, in two portions—one to cover in the chest of the patient, wider than the other, and capable of being slid backwards and forwards, by means of a sort of grooved joint, along the top of the bath, but fixed *in sitû* when the other, the foot half, is put on. When in position, the lid is secured by fastenings on the sides of the bath, and the patient becomes a helpless prisoner, his head only appearing above the lid, through an aperture in it formed by cutting out a portion sufficiently large to give room for the neck, but too small to draw the head through. In fact, except that the position is horizontal, and the hands and feet are not passed through holes, the patient occupies as secure, and not a totally dissimilar position to a culprit in those more elaborately-constructed stocks contrived by our forefathers, in which not only the hands and feet were made fast, but the neck also secured in their grip, leaving the helpless head to dangle in its front.

These covers, or lids, are chiefly employed when the douche or prolonged bath is employed: we should, however, rather see them dispensed with in the former case, when violence or resistance and excitement have to be allayed; for the act of placing a violent patient in a warm bath is often difficult, and must be more so where additional apparatus is to be applied. In short, the whole plan, especially the forcible mechanical restraint, is calculated to arouse irritation, and to produce an impression of the exercise of undue severity. On the other hand, the administration of a prolonged bath almost necessarily involves confinement of the body within an enclosed bath, in order to secure its continuation for many hours. Each bath is perforated at the bottom in three places—one for the entrance of hot, another for that of cold water, and the third for a waste-pipe. This last is furnished with a valve, opened by thrusting downwards against it an iron rod, and consequently intended to be under the control only of the attendant. The baths and the bath-rooms were well kept.

The arrangement of several baths in one room, unless screens or partitions are interposed between them, is open to objections on the score of delicacy; objections, by the way, of less weight in France, where the circumstance of man and wife using together the same bath is not uncommon. Some disadvantage, too, is likely to result from the plan, by extending excitement to others around, when an obstinate or refractory patient is to be placed in a bath; and again, it must be an odd sight to see two or three patients soaking in their prolonged warm baths for several hours together,—and we cannot imagine the feeling of company, under the circumstances, to conduce to the allaying of the excitement or irritation the bath is intended to secure.

Compared with reference to the conveniences provided in the asylums of our own country, where every want of the inmates is, as far as possible, anticipated and liberally met, the Asylum of Marseilles appears deficient; yet a rigorous comparison is scarcely just, where the differences in the habits and feelings of the people, especially among the lower classes, are in many respects so marked, and the wants and sentiments of the one people are scarcely recognised or felt by the other. This remark applies to many adjuncts to cleanliness and decency, and to feelings of moral propriety, considered of primary necessity on this side, but almost ignored on the other side of the Channel. For instance, the fact of several persons bathing together in the same room, as just cited; the absence of those conveniences for washing the hands and face—of towels, basins and soap—always ready for use—everywhere found in the lavatories of British asylums; and the comparative deficiency, the rudeness and uncleanliness, of water-closet accommodation. This last in the Marseilles Asylum was particularly defective, as compared with that provided for in our institutions. On the dormitory floor there was no closet, but the wants of the inmates at night were provided against, as before stated, by a "commode" placed in each ward during the night, and removed by day. On the ground floor a single closet was constructed, apparently in each division, consisting of a perforated stone slab, very little elevated above the floor, not intended to be sat upon, but to be used *more Francorum;* unprovided with water to flush it, and placed immediately over a cesspool. Such a construction, however repugnant to English notions of decency and cleanliness, is after the fashion of the country, and doubtless our English water-closet would, for a long time at least, be unappreciated, and an object of much abuse.

The exercising court for each division occupies the square area, surrounded on three sides by the building, and open on the fourth towards the general garden of the asylum. It is necessarily of very contracted size, insufficient for the recreation of the patients, wanting in cheerfulness, and affords only a limited prospect in one direction. The very excellent scheme of a sunk fence was adopted to separate the court from the external grounds; had this been unthought of, the only usual place for the patients' exercise would have presented the appearance of an enclosed prison court. Most of the courts were planted with shrubs and flowers, which imparted as cheerful an appearance as could be given them. The flower-beds were surrounded by a light rustic fence, not offensive to the eye, but yet, we believe, unnecessary for the preservation of the plants, and might be dispensed with advantageously.

The kitchen occupies a central position, at the posterior part of the large court interposed between the male and female sections of the institution, with each of which it is brought into communication by a covered way, or corridor, entering it on either side. The male and female attendants do not come into contact with each other in the kitchen, but have the food distributed to them through a serving-window placed alongside the doors, entering from the respective corridors. Internally the kitchen is spacious, and of ample elevation. At its centre is the large cooking apparatus, consisting of hot-plates, ovens, boilers, &c., surrounding the central fire or furnace, whereby fuel is much economized, and the facilities of cooking wonderfully increased. Except that this description of cooking apparatus renders roasting impracticable, it seems to combine, in every other respect, great advantages; for it never annoys by its smoke and dust either in the apartment or on the food; it obviates the glare and scorching of the open kitchen-range; it presents a most extensive surface for cooking over; it is very clean; and last, not least, it is much more economical. This fashion of the cooking-stove is finding its way gradually in this country, but it will take some years yet to make it general; for the majority of our cooks are a prejudiced and imperious race, and rule with a high hand in their own domain of the kitchen,—and possibly, also, they may be enamoured of that fiery complexion so characteristic of the craft, due, doubtless, very much to the glare of their open ranges.

Behind the kitchen is a boiler-room, whence hot water for culinary purposes and for the baths is derived. This room serves likewise as a scullery. Above it is the large water-tank of the establishment.

The wash-house and laundry are two detached buildings on one side the asylum. The arrangements of the wash-house followed the usual French plan, by having a large central tank of water, and on each side a narrow space, about three feet wide, separated from the rest by a bar. The usual washing-boards, upon which the clothes are rubbed and beaten, were fixed on the outer side, but so low down that the patients had to kneel at their work. This is clearly a grave error, for it involves wetting the clothes about the body and legs; and the position itself is highly to be reprobated, the more so as inflicted upon patients.

The clothes underwent a preliminary process of boiling in some large oval coppers, with raised metal covers, fixed against the wall. A large drying-court adjoined the wash-house, and sufficed without recourse to drying-closets and in-door stoves,—thanks to the southern climate of Marseilles.

The linen and clothing stores occupied another building, and consisted chiefly of one large room, surrounded by shelves, partitioned into small compartments, and screened from dust by curtains. This apartment was in a state of beautiful order and cleanliness.

The chapel is central, being placed in the rear of the officers' quarters, and having a corridor of communication with each half of the asylum. It is small, and not calculated to accommodate more than fifty or sixty of each sex. The space occupied by patients is set back on either side of the high altar, and cut off from the central area by a latticed partition. Each recess has a gallery, in which the female inmates have their place, whilst the men occupy the ground-floor. Chairs are used to sit in, and the whole building is fitted up and decorated in the style usual in Roman Catholic countries.

The land belonging to the asylum is laid out as a flower and kitchen-garden, and suffices to supply the institution with vegetables and fruit. It is kept in very good order by the labour of the patients.

At the period of my visit there were about 640 patients in the asylum, the two sexes being in nearly the same proportion. Alterations and additions were still going forward on the male side, which, when complete, would increase the total accommodation. They were after the designs of M. Aubanel, the chief physician of the asylum, whose name is known in the profession as joint author, with M. Thorre, of a work on the statistics of insanity, based on the experience of the Bicêtre, near Paris.

Besides M. Aubanel, there are an assistant-physician (M. Sauze), two "internes," an apothecary, and a non-medical director. This last-named officer is a sort of steward, and is charged with the household details of the establishment, with the expenditure, and with the direction of the works, &c. The "internes" hold office for three years, and are under the direction of the physicians, acting as dressers, clinical clerks, and generally as assistants. The physicians visit at least twice a day, and are attended by their "internes."

The attendants upon the female patients are of a religious

Order, the "Sisters Hospitallers," and are under the control of a superior in the asylum, and the more remote but higher authority of the head of their order. Complaints against them are reported to the superior, who has the power of removal. These nurses are uniformly dressed in dark blue gowns, with broad, white, plain collars, and those remarkably eccentric, magnified caps which, by some vagary in his notions of millinery and fashion, the reverend founder of the Order has imposed upon them. The crucifix is an invariable appendage, along with its string of beads, and the more useful scissors.

The male attendants are of no Order, wear no uniform, and are dismissable by the chief physician.

The common dietary consists of three meals:—the first, at seven A.M., of chocolate and bread; the second, at eleven A.M., of soup and bread; and the third, at five P.M., of meat, vegetables, and bread, with an allowance of wine and water. Extra allowance is granted to those engaged in active work, and the sick are fed according to their requirements, under the supervision and control of the physicians.

Occupation is encouraged as much as practicable, and the patients' labour produces everything required for the asylum, except bread, which is procured from the town. Those patients engaged in work are employed between five and six hours a day, a considerable interval for rest and recreation being allowed in the middle of the day. Throughout the workshops and workrooms there was quiet and order, and much credit was due to the whole management of the institution for the state the wards and the inmates at large presented.

Unfortunately, I did not gather from the "interne," who most courteously conducted me over the asylum, the amount of information I could have wished respecting medical treatment. Prolonged baths are resorted to in cases of maniacal excitement, and the douche especially, as a means of repression, or as a punishment. Mechanical restraint is little employed, and only when deemed absolutely necessary; rarely any other means except the camisole is employed. Bed sores are rather common and severe—a circumstance indicative of inefficient nursing, and partly accounted for by the absence of water-cushions, or beds, to meet the wants of the bed-ridden, feeble, and paralytic patients.

From the scanty statistical details of the Marseilles Asylum in our possession, we find that, according to Raymond (as cited by Esquirol), there existed 96 inmates in the old institution in 1769; 121 in 1811, of whom 70 were males, and 51 females; and that between 1797 and 1818, 696 were admitted—viz., 345 men, and 351 women. In 1841 the number had augmented to

336; and according to official returns made in 1850, there were 444—206 male and 238 female patients. Lastly, in 1853, the total reached was 640.

These figures show a very rapid increase in the number of insane, or rather of those brought under treatment, particularly between 1841 and 1853. In the nine years been 1841 and 1850 the augmentation was not on nearly so rapid a scale as that between the latter date and 1853.

Upon a general review of the Marseilles Asylum, it must be admitted to be in a very satisfactory state; the patients appear well cared for, well nourished, and well clothed,—yet more, apparently, might be done for them, by supplying them with increased means for recreation and amusement, by allowing them liberty beyond the walls, by adding to the internal comforts—the furniture and fittings of their sitting-rooms—and generally by attention to those little details which tend to divert, instruct, and solace the mind under the unavoidable evils of seclusion from their homes and from society at large.

Looking to the building itself, it may be remarked generally that, whilst exhibiting many features of merit, it has those inherent disadvantages of its system of construction—that of enclosed square courts, which certain conveniences it possesses for management and classification cannot compensate. This is not, however, the place to examine the relative advantages and disadvantages of this or any other system, our business being the description of existing asylums, and not the consideration of the principles of asylum construction.

### THE ASYLUM AT MONTPELLIER.

Montpellier, the capital of the department of the Hérault, and the seat of one of the three Universities of France, is particularly famous for its medical school, founded in the thirteenth century. From the period of its foundation until the present century, this school exercised an immense influence on the theory and practice of medicine; its professors and graduates were sought after throughout Europe, and brought to it fame and prosperity, whilst Paris was unknown except for the study of the scholastic philosophy. However, during the past century, the medical school of Paris has risen to eminence, whilst that of Montpellier has progressively declined; and although the latter has produced from time to time several eminent men, not a few of whom have migrated to Paris to share in its rising greatness, yet it has ceased to occupy a prominent position among the medical institutions of Europe, and has taken the rank only of a provincial university, almost entirely limited to the training of physicians for its own and the adjoining departments.

Unluckily, too, it has become jealous of its successful rival, and loses no opportunity of detraction; rejects its medical doctrines, and ignores as far as practicable the talents of its professors. At the same time, in its own small way, it loudly sounds its own praises, glories in calling itself the school *par excellence* of Hippocratic medicine; rigidly adheres to the hypothesis of vitalism, and the old doctrine of fluxions, and appeals solely to experience for its guide. On the other hand, it denounces as useless, or otherwise lightly esteems, minute pathological research, pathological chemistry, microscopic investigations, the applications of comparative anatomy and physiology, and was foremost in decrying the numerical method of the illustrious Louis.

The reader will not be surprised that such a school should decline; that one wilfully setting aside as useless all the appliances furnished by modern science and progress for the more complete investigation of medicine, and for placing it on a sure basis, should cease to influence the medical doctrines of Europe.

The importance of Montpellier as a university town and an important medical school, might naturally lead one to expect its medical institutions, and among them its asylum for the insane, to rank among the best in France. However, this is by no means the case; for the benumbing influence of indifference to modern progress has operated to the prejudice, and to the comparative neglect, of the wants of the insane.

The existing asylum is deficient in almost all those conditions which are now all but universally held essential to the well-being and the treatment of lunatics, and presents numerous faults, both in structural arrangements and internal management, which, alas! there appears little immediate prospect of seeing amended.

It is situated in the suburb of the city, very near to the Medical School and to the Botanic Garden, and constitutes a section of the Hospice for old paupers and venereal cases: it is likewise, as in similarly associated asylums, under the same administration, has the same general offices, the same chapel, kitchen, laundry, dispensary for medicines, stores, &c., and receives provisions, contracted for in the town, through the hands of the same steward. Moreover, the connexion of the two is so intimate that many of the dormitories for the insane are no other than wards of the hospital.

As the asylum of Montpellier can in no respect be put forward as a model, the description of its plan of construction need only be brief. The oldest portion, specially constructed for the care of the insane, was commenced, on the male side, in 1821, according to Esquirol's model of a square court, surrounded by a row of single rooms, or cells, of only one story, except at the angles, where there is a first floor. In 1824, a similar division was prepared for the females. These square courts are completely enclosed, are entered by an open iron gate from one side, and surrounded by a covered corridor, supported on clumsy, thick, square stone columns. The floor of the corridor is, moreover, raised about fifteen inches above the level of the open court, and is covered with asphalte or paved with stone. The enclosed bare court necessarily offers a very restricted and most prison-like place for exercise. Each cell is about 12 feet square and 10 feet high; it is entered directly from the corridor, and lighted by a small window about 1½ feet by 2 feet, placed on one side the door, unglazed, well barred, and defended by an external shutter of wood, kept closed during the night, and at such other times as thought fit. The floor is paved with stone, and the stone walls whitewashed. The furniture consisted of a heavy thick wooden crib, fastened to the floor; this was filled with straw to constitute the bed of the inmate, who, if clean, had an upper and under sheet, but if not so, only the former, and then lay in the loose straw of his trough-bed. A coarse woollen coverlet and blanket above completed the bedding furnished for ordinary use to the patients in this, the refractory division. Each cell bears a number, engraved on a small brass plate inserted in the wall above the door.

The very objectionable construction of these sections being recognised by the medical authorities, they are used for only the worst of the refractory and noisy cases, as a constant residence, and consequently are not commonly fully occupied. Still, I saw two or three cases confined to their beds in these miserable rooms, on account of bodily ailment.

The first addition, in 1823, to the section described, was that of a range of one-storied buildings, containing a dining-room, a *chauffoir*, or room for exercise, furnished with a stove and a dormitory. The dining-room is a long apartment, with a double line of tables and benches, leaving between them a central avenue. The tables are of wood, stained and polished, and both they and the room at large were kept in very good order. The floor is paved, as in all other rooms; the walls whitewashed and bare; and the windows insufficient to thoroughly and cheerfully light the apartment. The drinking vessels are of pewter; knives and forks are allowed to all, save those considered particularly untrustworthy. All the patients are brought together at meals, except those who are refractory, and require to be served in their own rooms. Contiguous to the refectory is a small serving-room, where the food is received from the kitchen preparatory to its distribution. The *chauffoir*, intended as a day or exercising room, particularly for the more troublesome and the dirty inmates, is a long, dreary, bare room, with whitewashed walls, stone floor, and a rude bench fixed against the wall. We conclude that its name is derived from the existence of a stove near its centre—a useful appendage, certainly, in cold weather, of which Montpellier has a fair share, especially from the prevalence in winter and spring of cold, biting north-east winds, but rendered as objectionable as possible in appearance by the construction around it of an iron cage or guard, formed by stout angular iron bars, and of very considerable height. The windows are placed along one side only, and are securely barred with upright iron bars. Beside the fixed bench, the only other article of furniture was one of an unprecedented character in my experience. It was a stout, wrought-iron arm-chair, fixed firmly to the floor, near the stove, having its framework pierced here and there with round and square-cut holes, for the passage of cords and straps to confine the arms, legs, and waist of its occupant. The appearance of such a contrivance of itself indicated that, in the conception of its inventor and patron, strength and mechanical force were primary principles in the management of the insane. Although we were most pleased to learn that this chair had not been put in requisition for some few years, yet the pleasure would have been greatly enhanced had we heard of its expulsion altogether from the asylum, and of it transference to some museum of barbarisms of the dark ages. If unused and thought unnecessary, why was it retained? To this query we got no answer; it was there, and it seemed a trial to part with so ancient, well-tried, and energetic an appurtenance of the establishment. Perhaps it was held up *in terrorem* before the refractory; a utilitarian purpose much to be deplored.

A third section of the block of building in question is a large dormitory, having a row of beds on either side, whitewashed walls, and a stone floor. The windows are of sufficient size, of the French casement model, and barred with upright iron bars; at night the room is partially lighted by a single gas-jet. This dormitory is principally occupied by epileptic and dirty patients who are somewhat refractory; hence their beds and bedsteads, or cribs, resemble those provided for similar cases in the single rooms, and are equally unsatisfactory.

Besides the *chauffoir* and refectory rooms named, there is yet another attached to this division, viz., a small sitting-room, which has some pretensions to commonplace comforts and furniture. Those entitled to the benefit of this apartment were of the class of pensioners, and not the indigent. Here smoking was allowed, and amusement with cards and dominoes indulged in.

At another part of the asylum precincts, on the male side, was a one-storied outbuilding, containing a day-room for younger inmates, devoid of everything which an Englishman understands as comfort—small, bare, except of whitewash, and its only fittings a table and bench. Connected with it was a very small, confined, dull, damp, unpaved court, surrounded by other buildings, as little fitted as the day-room for its purposes, but otherwise calculated to engender despair, or to plunge into dementia its unhappy prisoners.

At this asylum there is a separate division for criminal cases—*détenus*—consisting of a few single rooms, a *chauffoir* or sitting-room, and a small general court. Two of the cells were of recent construction, and regarded as models. Each had a small window alongside the door, entering from the common court in front, and behind, in its back wall, a perforated metal plate, and another door opening outwards into a miniature court belonging exclusively to the cell. One great object of the back door was stated to be to secure a thorough draught and complete ventilation—an advantage, however, to be gained only when the cell is not occupied, or the patient is in the little back court. The window is closely barred, and further protected and darkened by a shutter outside. The ceiling is vaulted; the walls of stone, whitewashed; and the floor of asphalte, well laid. Each cell appeared from 12 to 13 feet by 10 feet in area, and 11 feet high. The idea of these new single rooms, each with its own little court, was undoubtedly derived from Auxerre, where it may be seen carried out much more completely. The common court in front of the row of cells was small, and nearly as unfit and wretched as that of the lads' quarter. In each cell the bedstead was fixed against the wall on one side.

Other apartments on the ground floor are, on the male side—the shops for carpenters, tailors, and shoemakers, and straw-plaiting, but they call for no special description; the only remark to be made being that the inmates were pretty well occupied, and the work carried on quietly and regularly. On the female side was a large, nearly square, well-kept work-room, in which it was gratifying to find some seventy patients, almost all engaged in needle-work, spinning, &c. Another pleasing feature in this room was the presence of some singing-birds hung in cages against the wall, in addition to the not unusual appendages, the crucifix and the Madonna. As if to neutralize any pleasurable effects that access to this the most comfortable room the patients possessed might possibly induce, the windows were barred in the usual fashion, and the stove surrounded by a cage 5 feet high, formed of stout iron bars, crossed diagonally, and painted of a brown colour. Surely the authorities of the asylum would be sorely puzzled to show cause for such supererogatory barring of common sitting-rooms on the ground-floor, where the occupants are constantly under the eye of one or more attendants, and their escape by the windows nearly impracticable. But the conservative principle of the medical school seems to haunt the asylum, and to stand in the way both of alterations and of innovations. This work-room served also, until the completion of the new wing, as the female dining-room. Its furniture consisted of some fixed tables, chairs, and fixed benches; it was well lighted by the windows, and when required could be moderately illuminated by two gas-burners.

Passing from the one-storied buildings of the asylum proper, we come to those portions of the hospice occupied by the insane, consisting of several wards used as dormitories, and one on each side—male and female—as the infirmary. This last-named apartment is on the first floor, and holds twenty beds. It is heated by a stove, about 4 feet high, placed in the centre, unprotected on the female side by a guard, although at parts its case had almost reached a red heat. The barred windows, furnished with outside *persiennes*, were tightly closed, and although it was winter, the room was disagreeably warm, and what was worse, the air was impregnated with the fetid vapours from the sick and foul patients; for infirm patients, whatever their habits, are brought together in the same ward. Respecting the room itself there is little to desire. Like the dormitories contained in the hospice, it was a fairly-proportioned room, of good elevation, with tiled floor, and, like them, was lighted at night by a gas jet. The bedsteads and bedding varied according to the character of the cases, some of which were paralytic, others epileptic and infirm, and others, again, labouring under some bodily disease of more or less severity, accompanied in a few with such mental excitement as was deemed sufficient to justify restraint by the camisole, or by fastening to a chair, where lying in bed was not enforced. The bedding for patients of dirty habits, and for epileptics, has been already noticed; for the clean there are a flock-bed, an upper and under sheet, a blanket, and a brown woollen rug or coverlet, in the infirmary; but in the ordinary dormitories this last was only of figured blue linen or cotton. The condition of the bedding was not everywhere satisfactory; in the infirmary some of the coarse woollen coverlets were ragged and patched, and the sheeting given to foul patients was exceedingly coarse and brown.

No bedsteads were constructed to meet the particular requirements of the paralytic, epileptic, and unclean inmates. Once, indeed, a something was tried for the epileptics—viz., the legs were nearly entirely sawn off from the common cribs, whereby the bed-bottom was brought down close upon the floor; the consequence was, the remedy was worse than the disease—dust, damp, and in some cases the urine, accumulated beneath, engendering dirt and bad smells, and the ill-conceived contrivance had to be given up. Now the epileptics, if dirty, sleep in a deep crib, of the ordinary make, among loose straw, into which they sink and become secure against falling out during a fit; if, on the other hand, they are clean, they are afforded the indulgence of an under-sheet to lay upon the straw, or, in rarer instances, have a less thick bed and paillasse than those supplied to cases uncomplicated with that fearful malady.

For those lost to notions of cleanliness and decency, nothing special was attempted: the patient had to repose in a heap of loose straw filling up the trough of his bed; whilst the disgusting practice prevailed of letting the urine run through the bottom of the bedstead on to the stone or tile floor beneath, where it would frequently remain until evaporation had diffused it through the air.

The dormitories for the generality of the inmates were in good order and sweet, and contained from twenty to twenty-five beds each, arranged on either side, not more than from eighteen inches to two feet apart, and leaving a passage up and down the middle of the room. They had the usual gas-lamp, but were not warmed. Unlike the provision made for refractory and dirty cases, the bedsteads were of iron, and the bedding was made up of a paillasse, a flock bed, a pair of sheets, a blanket or two, and a blue linen coverlet. At the foot of each bedstead was a chair. As remarked in our notice of the asylums of northern Italy, the custom of the people is to lie high at night; so it is in this part of France, for we find that the top of the bed, by reason chiefly of the thickness of paillasse and bed, is from three feet to three and a half feet above the ground.

All the patients, with the exception of the very troublesome and noisy, sleep in dormitories, where they are under the surveillance of one or two attendants who sleep in the same apartment with them, as well as of the night attendant who perambulates the several parts of the building. In the infirmary there is always an attendant on duty night and day. Night commodes are provided in the dormitories, but no water-closets. Before going to bed, patients needing such care are attended to, and are besides got up in the course of the night; a proceeding which has been found to answer in many instances, although the number of dirty patients at Montpellier still is considerable.

The type of water-closet is "homological" with that seen at the Marseilles Asylum, yet the actual condition of things is worse. Lavatories, soap and towels, are, as usual in continental public institutions, wanting; indeed, at Montpellier, not even a vessel of water with *robinet* was anywhere seen. For personal cleanliness, therefore, the only opportunity is to be found in the occasional use of the warm baths.

The bath-house, according to custom, is a detached building; one for each department. It is ill-built and defective; the floor of stone, badly laid, uneven, and consequently always wet. The baths themselves are massive stone constructions, and furnished with heavy wooden lids, securely fitted upon them when required by strong iron fastenings. The supply of water, hot and cold, is by two taps placed above the foot of the baths; the waste pipe leading from the bottom. The baths are placed in a row alongside the wall, and between four and five feet above them is a horizontal water pipe, from which a short leather tube is suspended above the head of each bath to administer a douche. By this arrangement every douche-jet has equal force, and that variety which many physicians think desirable is unattainable.

As before intimated, the general offices are common to the asylum and the hospice, and do not afford occupation to the patients.

On the male side there are four airing courts: of these we have noticed three; the remaining one is also small, although superior by being not only planted with trees, which are indeed seen in two of the others, but laid out as a small flower-garden. The vicinity, however, of some houses, renders a high wall necessary around it. But besides the courts, there is a good-sized garden, which gives employment to several patients in the cultivation of vegetables and flowers. On the female side are three courts and a small garden for their use. The refractory court is bare, but the others are planted with trees, flowers, and shrubs. The flower-beds are protected by a slight railing, painted green; which, however unnecessary, has the merit of not being offensive to the eye. By the arrangement of the several parts of the asylum, the garden is nowhere visible except from some of the upper dormitories, and hence one great advantage of an open space about an asylum is lost—viz., the prospect of something more than the self-same court hour by hour, or day by day.

To refer now to the principles of management and treatment. The asylum, as a mere section of the hospice, is under the same government. This consists of a board or commission, constituted of the chief government officials and some few gentlemen of the department. These are charged with the supervision of the mode in which the institution is managed, have entire control over its funds and over every item of expenditure (which must receive their approval), and they appoint officers and servants. Their ruling principle is economy, cheapness, and the curtailment of the expenditure. To this board is no doubt principally chargeable the existing unsatisfactory and discreditable state of the asylum; for in all instances where to avoid expense is the primary rule of management, improvements are not to be looked for. What has been, and sufficed, may still be and suffice,—appears to be the Chinese maxim by which the authorities of the Montpellier Asylum are guided, and the maxim is equally fruitful of mischief and inefficiency as with the "Celestials."

The local representative of the Board exists in the person of the Director, appointed by it, and armed with the chief authority in the entire establishment, both hospice and asylum; subordinate to the Director is the Physician of the Asylum, an office at present held by M. Cavalier; he has under him an interne, who retains his appointment for three years. The physician is required to visit the patients twice every day; the first and principal visit is made between eight and nine in the morning; the second, usually directed to cases requiring particular attention, and of brief duration, in the afternoon. The duties of the visiting-physician are by no means formal, for they include the direction both of the moral and medical treatment, and are, as we personally witnessed, very thoroughly performed by the present officer. In his morning visit he goes throughout the building, receives reports of all the occurrences in it since his last visit, prescribes medically for all patients requiring it, directs, and in the case of douche, often superintends the administration of the baths, regulates the classification and employments, orders extra diet where required, and revises the general dietary every week. He is not precluded from private practice, and the appointment is tenable with a chair in the university.

M. Cavalier has occupied the post of physician between four and five years; and prior to that period was the "interne." His experience, therefore, has necessarily been considerable in the treatment of insanity, and we can testify to the zeal he displays in it, and to his kind and conciliating manner to patients, especially to the melancholic. He is a stanch believer in the efficacy of medical treatment when resorted to sufficiently early in the disease, and on this account devotes much time and pains to the investigation of bodily disorder and to its therapeutical treatment. It is nevertheless to be regretted that M. Cavalier has not had opportunities of examining the construction and management of asylums where modern innovations have found favour, and supplanted the ancient belief in the necessity of coercion, of bars, of high-walled courts, and of those many other deprivations of the consolations and comforts of life. To do him justice, however, we must mention that he is not insensible to many of the deficiencies and grave faults of the asylum he superintends, and is most desirous to see reforms affected; but the economical (?)

tendencies of the committee form an apparently insuperable impediment to the realization of his wishes. The "interne" who was in residence during my stay at Montpellier, was M. Campaña, a Spaniard by birth, but educated at Montpellier to whom I must make acknowledgment for the civility and attention received at his hands. Since that time I am glad to hear he is in charge of the asylum at Avignon. The "interne" has furnished rooms, board, light and firing, and receives a small gratuity. He has to carry out, or see carried out, the directions of the physician, keeps the registers, and is responsible for the good working of the asylum in the physician's absence.

The attendants do not belong to a religious order. In proportion to the number of patients they are nearly as one to twelve. Both male and female attendants are clothed uniformly. The men wear a blue jacket, brown trousers, and a felt cap ; the women were neatly dressed, and did not rejoice in those marvellous caps, the exclusive and quaint appendages of the heads of those under religious vows, particularly the Sisters of Mercy of St. Vincent de Paul.

The patients admitted into the asylum are of three classes—viz., pensioners, paupers, and criminal lunatics (détenus). In 1854, their total number was 375—to wit, 260 males and 115 females. Among these, 10 men and 3 women were accounted paralytic ; epilepsy was equally prevalent in the two sexes, but was not a common complication.

A uniform dress was provided for the patients. The women were in general neat and clean in appearance ; but the men were many of them slovenly dressed, with their clothes ragged and in disorder. As might be expected, this happened chiefly among the refractory, for no specially contrived dresses were provided to meet the requirements of such, or of any other class of patients presenting particular symptoms.

The ordinary diet-table provides four meals a day. The patients rise at six o'clock, and at seven have a slice of bread with coffee, and an apple or some other fruit ; at eleven, soup with vegetables ; at four in the afternoon, a dinner of soup, bread, and vegetables, on three days in the week with meat ; and, lastly, at six, they receive a portion of bread. Wine diluted with water is allowed at their two chief meals ; latterly the quantity has been much lessened, owing to wine (even in this wine-growing district) having advanced to four times its customary price. Of the vegetable food taken, peas (of two sorts), harricot beans, and rice cooked in several ways, form the staple. The food is inspected by the physician or interne, and may be rejected by them if thought unfit.

Employment is as much as possible promoted. About one-half of the male patients are occupied in the garden and grounds of the institution, and in the workshops. The tailors' workshop was the best filled: in all the shops the greatest decorum and quiet were observed. A party of men is selected and entrusted to go out into the town and its vicinity to bring back requisites for the asylum use. Those are not chosen who belong to the city, and are acquainted with it and with persons living in it. On one day I saw a band of thirty, carrying hand-barrows, and proceeding in a sort of loose military order, under charge of three attendants; for none are ever allowed to be out by themselves. One attendant preceded them, whilst the other two brought up the rear. Moreover, about thirty male patients leave the asylum daily to work at some little distance in the country, at quarrying stone for building, in cutting timber, preparing wood, &c.

Of the women, two-thirds are employed in needlework, in house-cleaning, straw-plaiting, and the like female occupations.

A very complete record is kept of the occupations of the patients, showing the nature of the employment, the length of time engaged in it, the manner in which it has been undertaken, whether with alacrity or unwillingly, and other similar particulars.

Little is done in this asylum to provide recreation or amusement for the inmates. Excepting dominoes and cards in one section, I did not see any other pastime. Books and papers, if allowed, are extremely scarce, and not provided by the institution; the reunion of patients at concerts, dances, lectures, and the like, has not been contemplated, and out-door amusements are out of the question from want of space.

Novelists are fond of representing the peasantry of South France as given to revelry and perpetual gambollings to the sound of the pipe; and this might be true of them in the olden time under their good king René, but it is purely mythical of the present generation, which is sufficiently dull, heavy, and ignorant. They retain their ancient language, the Provençal or Langue d'Oc, and are many of them unacquainted with French. It would be well were the schoolmaster abroad in this portion of France; indeed, it would be a happy thing could he penetrate into the Asylum of Montpellier, and diffuse a better knowledge of the moral treatment of the insane, illustrated by the practice in other parts of Europe.

There is no chapel for the exclusive use of the asylum. The patients attend once every Sunday at the chapel belonging to the hospice; and they are visited by a priest from time to time, or when his ministrations are called for to the dying: his visits being under the control of the physician.

The patients are allowed to see their friends, under the sanction of the physician, twice a week, and have a room set apart for the purpose. An attendant is always present during the visit, and is expected to render an account to the medical officer of the behaviour of the patient, of the conversation, of inquiries and complaints made, in short, of all that has passed.

To come, now, to details touching the medical treatment. Restraint is largely used, notwithstanding the principle professed is to employ it only where absolutely required; but, as usual, the sanction of its employment affords a loophole for its frequent use; the indolent or the unmerciful, and the attendant without forethought and readiness, or measures of expediency, will discover sufficient reason for its adoption, and be ever ready to justify it even where its application must be reported to the superintending physician. At Montpellier the ordinary means of coercion is by the camisole, and occasionally by belts and straps to confine the limbs or the trunk. The rule with homicidal criminal patients seems to be to keep them constantly confined: one such male always wore a camisole when not shut in his room, was regarded with fear, and was no doubt led, by his treatment, to believe himself a very formidable character, and to endeavour to prove his ferocity when he got a chance of doing so.

On the occasion of one visit to the female side of the house, I noticed seven women wearing the camisole in the refractory ward, and one in the work-room; another was held in a clumsy wooden arm-chair by a piece of stout tape loosely tied around her waist, which could indeed serve as a means of restraint only to her morbid apprehension. In the infirmary a few were restrained by camisoles, and some others confined to chairs constructed to allow the escape of the excretions from their persons; a beastly contrivance, too common, we are sorry to say, in French asylums. I was assured that none were fastened in bed at night, except under very extraordinary circumstances.

The douche is exclusively employed as a punishment, and as such is in pretty frequent requisition. It is not used as a therapeutical agent, but only for those who are refractory and violent, or who refuse to work or to take food. With the last object in view it was represented as very successful, and that forcible alimentation by the œsophageal tube had not been resorted to for three years, although refusal of food had been a circumstance of no uncommon occurrence. The duration of the douche is regulated by the physical condition of the patient; and its application is always made under the superintendence of the physician, or of his assistant, the "interne." It is sometimes repeated in the course of the same day. The vertical fall of the stream is, however, as before stated, not more than four feet, and its shock, therefore, is not very great.

Prolonged baths are frequently used in acute mania to allay excitement and to induce sleep. They are unsuited to weak patients, and their effects require to be diligently watched. According to the severity of the case and the strength of the patient, they are continued for ten, twelve, and even for twenty-five hours. Food and medicine are administered during the bath, and in some instances a thread of cold water is allowed to trickle over the shaven head.

The selection of medicinal agents is regulated entirely by the physical symptoms of the patients, and no general mode of treatment is pursued. Opium and morphia are very seldom given, for there is a hypothetical objection to them prevalent in this part of France, as well as in Italy—viz., that they cause a revulsion to the head, and accelerate the onset of dementia. In lieu of opiates, therefore, conium and hyoscyamus, along with their active principles and preparations, are employed.

The question of the relative advantages of dormitories and single rooms has been much discussed in this country, and strong opinions expressed; the majority, we think, against dormitories, except for a comparatively small class of patients. We shall see, in the course of our examination of foreign asylums, the use of dormitories for almost all, in some places indeed for all, patients. Here at Montpellier, some twenty-four cells were provided in each division—male and female; all the other sleeping accommodation was in dormitories. But, in fact, this proportion of single to associated sleeping-rooms did not exist, for all the cells were not occupied, and had not the demand for admission been so great that room in dormitories could not be given more largely, still fewer single rooms would have been used.

M. Cavalier's opinion was decidedly in favour of dormitories for the whole population, with very few exceptions; and as the result of his experience, he stated that it is rare that one patient disturbs another; that, on the other hand, he has found those who were restless and noisy by themselves in single rooms, become quiet when placed in a dormitory. Even epileptics do well in dormitories—a class of patients which many who recognise the utility of common sleeping-rooms over single ones, have a strong feeling against their association together at night. M. Cavalier has never known an accident or ill consequence occur in the epileptic dormitories, even where an access of furor has overtaken one of the inmates. In such instances, indeed, speedy attention is required; and this is ensured by the presence of attendants in the apartment, which is always secured. Moreover, the physician mentioned would have no dormitory to contain less than twenty beds, and approves of the association of even larger numbers.

Upon a review of the preceding account, we may observe that the asylum at Montpellier is to be condemned on account of its site, its too limited area for exercise and employment, its confined, high-walled, and enclosed courts, its most faulty construction, its connexion in general administration with the adjoining hospice, its deficiency in almost everything to solace its unfortunate inhabitants, its management by a non-resident officer, and in the paramount control exercised by non-medical men. We need not sum up the remarks on the internal management, medical and moral; yet we are desirous to testify to the attention given to the former, and should, on the other hand, be glad to awaken an increased interest in the moral and physical state of the asylum occupants. The women's side of the house was better kept than that of the men; but everywhere the dormitories were clean, and orderly, and sweet, with the exception of the infirmaries. The presence of a few birds and domestic animals about the day-rooms; of pictures, flowers, and ornaments in general; would much enliven the abode of the poor lunatics. Moreover, the construction of some decent water-closets, of lavatories, or of some means to promote personal cleanliness, the provision of a few articles of furniture, the introduction of periodicals and books, and of other means of amusement, would render the present most ill-adapted building a somewhat less objectionable residence for the insane than in its present state it is.

An attempt to adapt the present structure to its purpose would be fruitless, and a waste of money. This fact is so obvious, that the authorities of the asylum have determined to erect a new one; yet, it will be read with astonishment, their intention is to retain the same site, so little knowledge have they of what is requisite to the well-being and recovery of the insane. The new edifice was already in progress when we stayed at Montpellier, and a new wing, in rear of the existing asylum, was considerably advanced. This was a two-storied structure, of stone, and certainly an improvement upon the old building. As it was designed to relieve the present asylum, which could no longer meet the wants of the department, its accommodation was particularly planned to supply existing deficiencies, whilst it should at the same time harmonize as a section of the projected new buildings. On the male side, therefore, it would furnish a billiard or amusement room; and an infirmary specially for dirty cases, on the ground floor, and above, additional dormitories. On the female side, the similar extension of the asylum would supply an infirmary and work-room on the ground floor, and dormitories above. The precautions for the safe custody of the inmates are unluckily to be repeated, and the windows to be guarded by iron bars; with this improvement, however—that the external bars are to correspond in figure and position with those of the casements, so that they may be unperceived when the latter are closed.

The retention of the same site for the projected new asylum, entails the perpetuation of many of the worst evils of the present establishment, and cannot be too strongly deprecated by all who are interested in the welfare of the insane. The site is objectionable by its proximity to the town, and its immediate vicinity with a low suburb; by its limited area, which will never admit of sufficiently extensive airing courts and ground for employment, and by its involving the continuance of the prejudicial connexion between the hospice and asylum.

Some excuse may be found for the faulty position of the Venetian asylums, since for these no other and better can be found except on the distant mainland, a removal which would cause not only great immediate cost, but always occasion difficulty and much expense in the carriage of patients. But here at Montpellier, the authorities have on every side numerous most eligible positions for the erection of an asylum, with all the advantages of greater healthfulness, of ample ground for cultivation, and of fine and cheerful prospects, in short, of all those external conditions calculated to divert, to alleviate, and to heal the disordered mind.

It would have added interest and value to this history of the Montpellier Asylum could we have appended the statistics for some years past; these, however, were not obtainable, not being published, but buried among the departmental archives. The only details in our possession belong to the four years from 1829 to 1832 inclusive, and are given in an excellent record of the clinique of M. Rech, a former physician of the asylum, to whom Esquirol attributes the high merit of having "overthrown the fatal prejudices against the insane in a country where some regarded them as outcasts from God, others as the peculiar favourites of heaven, and where all agreed in believing them incurable."

According to the statistics referred to, there were resident in the asylum at the commencement of 1829, 57 men and 50 women, in all, 107; admitted in the four years between 1829 and 1832 inclusive, 89 males and 52 females, together, 141. During those four years there died 27 men and 19 women, or 46 in all; were discharged cured, 23 men and 19 women, together, 42; and uncured, 13 men and 6 women, in all, 19. The proportion of deaths in the two sexes, taken in relation with their relative number, is 1 to 13 among the men, and 1 to 15 among the women. The ratio of cures, calculated upon the admissions, was about 30 per cent.; and during the four years in question, was greater among the females than the males. Considered with reference to the form of the malady, there were of the 107 existing at the beginning of 1829, suffering from—

| | |
|---|---|
| Mania | 24 |
| Monomania | 13 |
| Dementia | 49 |
| Idiocy | 8 |
| Intermittent mania | 2 |
| Epileptics | 11 |
| | 107 |

Of 151 admissions, there were suffering from—

| | |
|---|---|
| Mania | 40 |
| Monomania | 36 |
| Dementia | 45 |
| Idiocy | 12 |
| Intermittent mania | 8 |
| Mania with delirium | 1 |
| Epileptics | 9 |
| | 151 |

Among the cured were—

| | |
|---|---|
| Mania | 23 |
| Monomania | 11 |
| Dementia | 8 |
| | 42 |

There died from—

| | |
|---|---|
| Mania | 6 |
| Monomania | 4 |
| Dementia | 24 |
| Idiocy | 1 |
| Epilepsy | 11 |
| | 46 |

The assigned causes of death were—

| | |
|---|---|
| Cerebral affections | 21 |
| Diarrhœa | 9 |
| Phthisis | 9 |
| Slow fever | 4 |
| Contagious fever | 1 |
| Pneumonia | 1 |
| Pleurisy | 1 |
| | 46 |

In 1835, the population, as Esquirol records, had reached to 158—viz., 75 men and 63 women. In 1825, it was only 75, but in the course of the three following years there were 106 admissions.

The most complete statistics of an institution are liable to engender fallacies, and, in the opinion of some, can be made to prove anything; but whatever the defects of statistics may be when sufficiently extended, those few which we have been able to obtain of the Montpellier Asylum are certainly insufficient to draw any general inferences from, which have value enough to make them worth a place in this paper.

---

